# Cardiac force-frequency relationship and frequency-dependent acceleration of relaxation are impaired in LPS-treated rats

**DOI:** 10.1186/cc7712

**Published:** 2009-02-06

**Authors:** Olivier Joulin, Sylvestre Marechaux, Sidi Hassoun, David Montaigne, Steve Lancel, Remi Neviere

**Affiliations:** 1 EA 2689, IMPRT-IFR114, Université de Lille 2, 1 place de Verdun 59000 Lille, France; 2 Service Explorations Fonctionnelles Cardiovasculaires, CHRU Lille, Bd Pr. Leclercq 59000 Lille, France; 3 Département de Physiologie, Faculté de Médecine, 1 place de Verdun 59000 Lille, France

## Abstract

**Introduction:**

Frequency-dependent acceleration of relaxation (FDAR) ensures appropriate ventricular filling at high heart rates and results from accelerated sarcoplasmic/endoplasmic reticulum calcium ATPase (SERCA) activity independent of calcium removal from the cell. Because lipopolysaccharide (LPS) challenge may induce aberrations in calcium trafficking and protein phosphorylation, we tested whether LPS would abolish FDAR in rats.

**Methods:**

Following LPS injection, changes in force-frequency relationship and FDAR were studied in cardiomyocytes, isolated hearts and *in vivo *by echocardiography. Calcium uptake and phosphatase activities were studied in sarcoplasmic reticulum (SR) vesicle preparations. Western blots of phospholamban and calcium/calmodulin-dependent protein kinase II, and serine/threonine phosphatase activity were studied in heart preparations.

**Results:**

In cardiomyocytes and isolated heart preparations, reductions in time constant of relaxation (τ) and time to 50% relaxation at increasing rate of pacing were blunted in LPS-treated rats compared with controls. Early diastolic velocity of the mitral annulus (Ea), a relaxation parameter which correlates *in vivo *with τ, was reduced in LPS rats compared with control rats. LPS impaired SR calcium uptake, reduced phospholamban phosphorylation and increased serine/threonine protein phosphatase activity. *In vivo *inhibition of phosphatase activity partially restored FDAR, reduced phosphatase activity and prevented phospholamban dephosphorylation in LPS rat hearts.

**Conclusions:**

LPS impaired phospholamban phosphorylation, cardiac force-frequency relationship and FDAR. Disruption of frequency-dependent acceleration of LV relaxation, which normally participates in optimal heart cavity filling, may be detrimental in sepsis, which is typically associated with elevated heart rates and preload dependency.

## Introduction

Apart from the Frank-Starling mechanism, force-frequency relationship represents a major intrinsic regulatory factor that is essential for the immediate adjustment of cardiac contractile function to rapid changing requirements of blood supply. The frequency-dependent gain in contractility is an intrinsic property of cardiac muscle present in all mammals and allows for greater contractile force [1]. Not only does the heart generally beat stronger when it is stimulated to contract faster, the kinetic of contraction is also accelerated, that is, the frequency-dependent acceleration of relaxation (FDAR) [1,2]. From a physiological perspective, FDAR participates in the maintenance of efficient ventricular filling and coronary blood at higher heart rates, despite a decreased diastolic time interval [2]. In clinical sepsis, left ventricle (LV) systolic dysfunction and altered diastolic relaxation are typically observed [3]. In contrast, only a limited number of studies have evaluated the frequency-dependent gain in contractility in the septic myocardium. In these studies, inotropic responsiveness to changes in frequency of stimulation from lipopolysaccharide (LPS) treated hearts was significantly less than controls [4,5]. Effects of LPS on FDAR have not been previously described.

Force-frequency relationship and FDAR are primarily related to changes in intracellular calcium transients [1,2]. The exact molecular basis for FDAR has not been resolved, yet an attractive mechanism implicates thr-17 phosphorylation of phospholamban by calcium/calmodulin protein kinase II (CaMKII) [6-8]. In addition to these intrinsic heart regulatory processes, stimulation of β-adrenoceptors increases contractility and accelerates relaxation through accumulation of cyclic AMP and subsequent activation of protein kinase A. Activated protein kinase A phosphorylates phospholamban at ser-16 residue that relieves sarcoplasmic/endoplasmic reticulum calcium ATPase (SERCA) inhibition, enhances removal of calcium from the cytosol and increased heart contractility [8]. Conversely, activation of protein phosphatase-1 and 2, which are the major phosphatases functionally relevant in the heart, dephosphorylate phospholamban and favour SERCA inhibition [8].

We hypothesised that intracellular calcium traffic aberrations and changes in calcium handling protein phosphorylation reported in LPS challenge [9-11] would alter FDAR response. For example, reduced phospholamban phosphorylation by protein kinase inhibition [10,12] and activation of protein phosphatases that dephosphorylate phospholamban [13,14] typically observed in sepsis may in turn alter inotropic and relaxation responsiveness with changes in frequency of heart stimulation.

The present experiment was undertaken to assess the potential effects of LPS on force-frequency relationship and FDAR in rats. Preparations of intact cardiomyocytes, isolated hearts and echocardiography were evaluated. First, we tested whether LPS would reduce phospholamban phosphorylation and disrupt cardiac force-frequency relationship and FDAR. As FDAR was disrupted in LPS-treated rats, we next tested whether phospholamban dephosphorylation induced by LPS was associated with CaMKII activation (which phosphorylates phospholamban at the thr-17 residue) and serine/threonine phosphatase activation (which dephosphorylates phospholamban).

## Materials and methods

### Animal preparation

All work was performed under a protocol approved by the University of Lille's Institutional Animal Care and Research Advisory Committee. The investigation conforms with the *Guide for the Care and Use of Laboratory Animals *published by the US National Institutes of Health. Under brief isoflurane anaesthesia, adult male Sprague-Dawley rats (weighing 250 to 300 g) (Charles River Lab, L'Arbresle, France) were treated with either 10 mg/kg of LPS from *Escherichia coli *serotype 055:B5 in 500 μL saline or 500 μL saline administered intravenously via the dorsal penile vein. Where indicated, we used tacrolimus (FK506; Fujisawa, La Celle St Cloud, France) as a protein phosphatase type 2 inhibitor. Tacrolimus-treated LPS-challenged rats received 0.01 mg/kg of tacrolimus in 500 μL LPS in saline mixture. Four hours after treatments, rats were prepared for echocardiography, and isolated heart or single cardiac myocyte evaluations.

### Left ventricular cardiomyocyte shortening

Ventricular myocytes were isolated as previously described [15]. For contraction amplitude, cells were placed in a flow chamber and field-stimulated with pulses of 5 ms duration at a frequency of 0.5 and 2 Hz. As an index of acceleration of relaxation, we calculated time constant of relaxation (tau, τ) at 0.5 Hz and 2 Hz.

### Myocardial in isolated heart preparation

Myocardial contractile function was studied using a modified Langendorff isolated heart preparation technique, as previously described [16]. After the equilibration period, heart parameters were recorded at 150 and 300 beats/minute pacing rates. Left ventricular developed pressure (LVDP), its first maximal derivatives (dP/dt_max _(positive) and dP/dt_min _(negative)) and coronary perfusion pressure were recorded using a Biopac Data Acquisition System (Biopac Systems Inc., Goleta, CA, USA). Half-relaxation time (t_1/2_) and time constant of LV isovolumic relaxation (tau, τ) were calculated at 150 and 300 beats/minute. LV pressure from the time of peak negative dP/dt to 5 mmHg above LV end diastolic pressure was fitted by the monoexponential equation:

p(t) = pe^-t/τ^

where t is time obtained, e is natural logarithm and p is pressure [17]. Time constant of LV isovolumic relaxation (τ) were calculated from the above equation.

### Echocardiography evaluation

Rat echocardiography was performed as previously described [18] at baseline and four hours after intravenous administration of LPS in the same individual. Two-dimensional (2D) Doppler echocardiography was obtained in the left lateral decubitus position with a linear transducer (14 MHz, Acuson Sequoia C512 system, Mountain View, CA, USA). All echocardiographs and data analysis were performed by MS, blinded for group design. Measurements were performed after magnification to ensure optimal visualisation of cardiac chambers, and depth was set at 20 mm. Gain was set for best imaging and compression was 65 dB. For the assessment of LV function, parasternal short and long axis 2D views were sampled to obtain at least 15 images per second. For blood flow and tissue Doppler measurements, the sweep speed was 200 mm/s.

The anterior chest hair was shaved off and recordings were made under continuous monitoring by fixing the electrodes to the limbs. At least three cardiac cycles were used for each measurement, and the average value was taken. M-mode tracing of the LV was obtained from the parasternal long axis view allowing the measurement of LV end diastolic diameter, LV end systolic diameter, and diastolic posterior and septal wall thickness in accordance with the American Society of Echocardiography guidelines. The following parameters were calculated: left ventricular weight = 1.04 × (LVEDD + PW + SW), and fractional shortening = (LVEDD - LVESD)/LVEDD, where LVEDD is left ventricle end diastolic diameter, LVESD is left ventricle end systolic diameter, PW is diastolic posterior wall thickness and SW is septal wall thickness.

**Figure 1:**
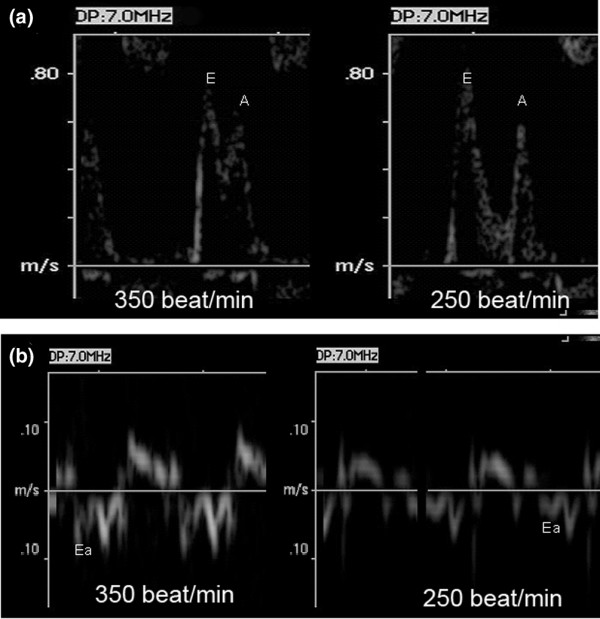
Representative spectral recording of blood flow Doppler and tissue Doppler imaging recorded at the spectral mitral annulus. **(a) **The blood flow Doppler was recorded at the tips of the mitral leaflets and **(b)** tissue Doppler imaging was recorded at the spectral mitral annulus in a normal rat at heart rate of 350 beats/minute and 250 beats/minute. Note that in the presence of minimal changes in early flow and late flow mitral diastolic wave velocities, early diastolic mitral annulus velocity is lower when heart rate is decreased.

From the parasternal short axis view, pulmonary flow was recorded using pulsed Doppler with the smallest sample volume placed at the level of the pulmonary annulus. Cardiac output was calculated as the product of the pulmonary forward stroke volume:

VTI × D^2^/4 × π

where D is the diameter of the right ventricle outflow tract, and heart rate, and VTI is velocity time integral. Pulsed Doppler mitral inflow velocities were obtained by placing a 0.6 mm sample volume between the tips of the mitral leaflets in the apical four-chamber view. The Doppler beam was aligned parallel to the direction of flow. Isovolumic relaxation time was measured as the interval between aortic closure and the start of mitral flow. Ea was obtained from the four apical chamber view using tissue Doppler imaging as an indice of LV relaxation. Data were stored on compact discs in DICOM format and measured offline with the Echo PAC PC Software release 08 (General Electrics, Horten, Norway). Transthoracic echocardiography was performed under inhaled sevoflurane anaesthesia, 100% oxygen and spontaneous respiration. Increases in sevoflurane concentrations (2 to 4%) were used to decrease heart rate by about 20%. An echo image is shown in Figure 1.

### Western blot analysis

Ventricular heart tissue was homogenised with a Polytron homogeniser (Glen Mills Inc., Clifton, NJ., USA). Protein extracts from heart tissue (50 μg) were separated by a 4 to 12% bis-Tris HCl-buffered polyacrylamide gel (Invitrogen, Carlsbad, CA, USA) and subjected to Western blotting for SERCA2a, phospholamban, thr17-phospho-phospholamban, CAMKII and phospho-CAMKII antibodies (Affinity Bioreagents, Golden, CO, USA). Bound antibodies were detected by the use of enhanced chemiluminescence's Plus kit (Amersham, Freiburg, Germany).

### SR vesicle calcium uptake

Sarcoplasmic reticulum (SR) microsomes were obtained from rat ventricles following ultracentrifugation (100,000 g) procedures [19]. The whole procedure was carried out in a cold room, at 4°C and in the presence of protease inhibitors (0.1 μM aprotinin, 500 μM benzamidine, 1 μM leupeptine, 1 μM pepstatin A 200 μM and phenylmethylsulphonyl fluoride). SR preparation was placed in a Teflon chamber equipped with a calcium-selective microelectrode (WPI, Aston, UK) to assess calcium-uptake activity. Changes of medium (ie, extramicrosomal) calcium concentration were recorded continuously. At the end of the preincubation period, the reaction was initiated by addition of 1.5 mmol ATP after which calcium chloride pulse was added. Calcium is then rapidly taken up by the SR vesicles, resulting in a return of extramicrosomal calcium concentration to baseline levels. At the end of the experiments, thapsigargin was added to block SR calcium uptake.

### SR phosphatase activity assay

SR protein phosphatase activity was assessed in SR vesicles of rat with the Protein Serine/Threonine Phosphatase Assay System (Millipore; Bioscience, St Quentin en Yvelines, France) according to the manufacturer's instructions [20].

### Statistical analysis

Results were analysed with the SPSS for Windows software, version 11.0.1 (SPSS France, Paris, France). Data represent means ± standard error of the mean. Statistical nonparametric Mann-Whitney test was used to compare unmatched groups (controls and LPS-treated rats). Statistical comparisons between means were made by two-way analysis of variance (ANOVA) for repeated measurements on frequency effect (main effects; two levels and treatment effect; two levels), and the interactive effects. Post hoc analyses were made using Dunnett's test comparing the variable group with the control group. Statistical significance was assigned to p < 0.05.

## Results

### Single cardiomyocyte and myocardial function

Shortening of single cardiomyocytes isolated from LPS-challenged rats was reduced compared with controls. Isolated heart-derived contractility and relaxation parameters, such as LVDP and its first maximal derivatives, were also reduced in LPS-challenged rats (Table 1). Echocardiography evaluation shows that LPS challenge induced about a 15% decrease in LV ejection fraction and about a 35% decrease in fractional shortening, compared with controls (Table 1). Decreases in indexes of cardiac performance were accompanied by about a 40% decrease in cardiac output. Ea was reduced in LPS-challenged rats compared with controls, suggesting perturbations of LV relaxation. Early flow (E)/Ea did not change significantly, suggesting minor modification in LV end diastolic pressure (Table 1). Overall, our results suggested that LPS induced some degree of hypovolaemia which was associated with LV diastolic dysfunction.

**Table 1:** Haemodynamic characteristics

	Control	LPS
Cardiomyocyte shortening	6.3 ± 0.4%	4.5 ± 0.4%*

LV developed pressure (mmHg)	90 ± 5	65 ± 7*

dP/dt_max _(mmHg/second)	2750 ± 100	1650 ± 175*

dP/dt_min _(mmHg/second)	1300 ± 215	800 ± 115*

Heart rate (beats/second)	379 ± 13	350 ± 21

LV ejection fraction (%)	62 ± 3	51 ± 8*

LV fractional shortening (%)	46 ± 4	29 ± 6*

Cardiac output (mL/minute)	110 ± 14	65 ± 20*

Transmitral E velocity (cm/second)	87 ± 13	61 ± 5*

Transmitral A velocity (cm/second)	68 ± 21	47 ± 11*

Early diastolic velocity Ea, (cm/second)	8.5 ± 0.9	5.3 ± 1.3*

E/Ea	10.1 ± 2.6	11.5 ± 2.7

Shortening was measured in single cardiomyocytes (20 cells per cell isolation, 6 rats per group). Left ventricle (LV) developed pressure and its first derivatives were measured in isolated heart preparations (8 rats per group). Heart rate, LV ejection fraction, LV fractional shortening, cardiac output, transmitral and early diastolic velocities were assessed during transthoracic echocardiography (5 rats per group). Results are expressed as means ± standard error of the mean and analysed by the mean of unpaired t test. * indicates p < 0.05 vs controls. A = late flow; dP/dt_max _= LV developed pressure first maximal positive derivatives; dP/dt_min _= LV developed pressure first maximal negative derivatives; E = early flow; Ea = early diastolic mitral annulus velocity; LPS = lipopolysaccharide.

### Force-frequency relationship and frequency-dependent acceleration of relaxation

**Figure 2:**
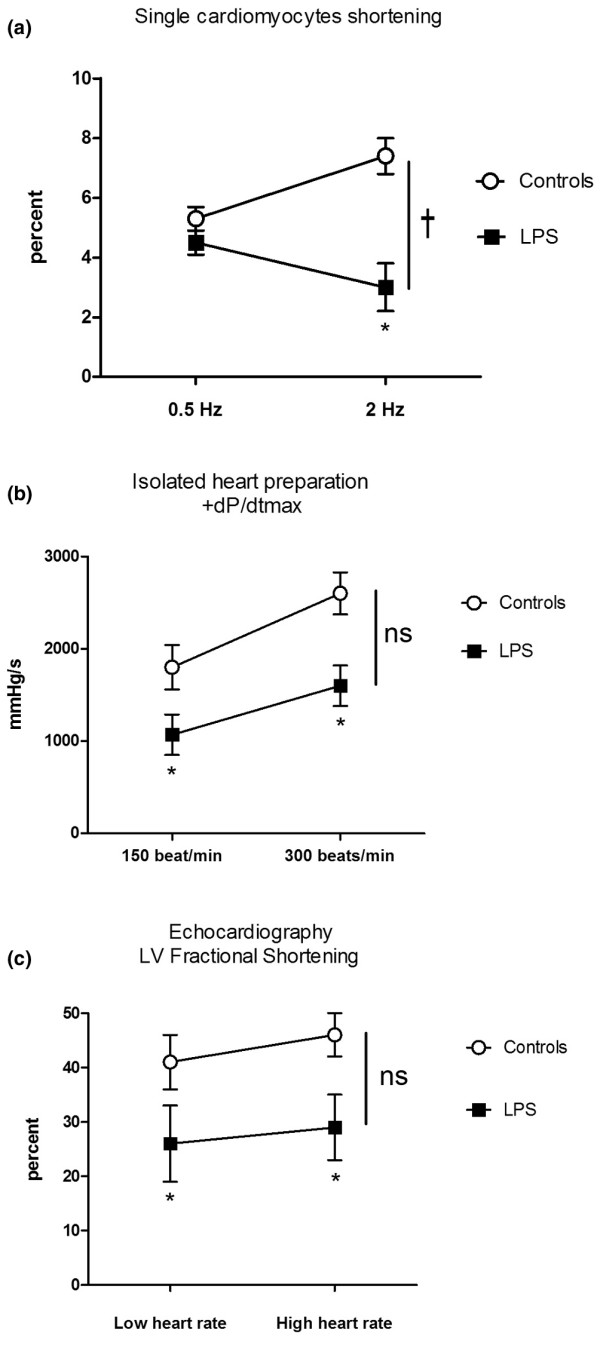
Effects of heart rate changes on contractile performance. This was measured in **(a)** single cardiomyocytes (n = 6 per group), **(b)** isolated heart (n = 8 per group) and **(c) **echocardiography (n = 5 per group) studies. Results are mean ± standard error of the mean; analysis of variance for repeated measurements on frequency effect, treatment group effect and the interactive effects. * p < 0.05 between control and lipopolysaccharide (LPS) at each frequency † p < 0.05 between groups across frequency. Overall, LPS resulted in significant different force-frequency dependence in cardiomyocytes, but not in isolated hearts and echocardiography.

In control cardiomyocytes, increasing rate of pacing from 0.5 to 2 Hz resulted in a positive cell shortening-frequency response, which was inverted in cardiomyocytes isolated from LPS-treated rats (Figure 2a). In contrast, similar positive force-frequency responses were observed in whole heart preparations, that is, isolated heart and echocardiography, from control and LPS-treated rats (Figures 2b,c). Overall, LPS resulted in significantly different force-frequency dependence in cardiomyocytes, but not in isolated hearts or *in vivo*.

**Figure 3:**
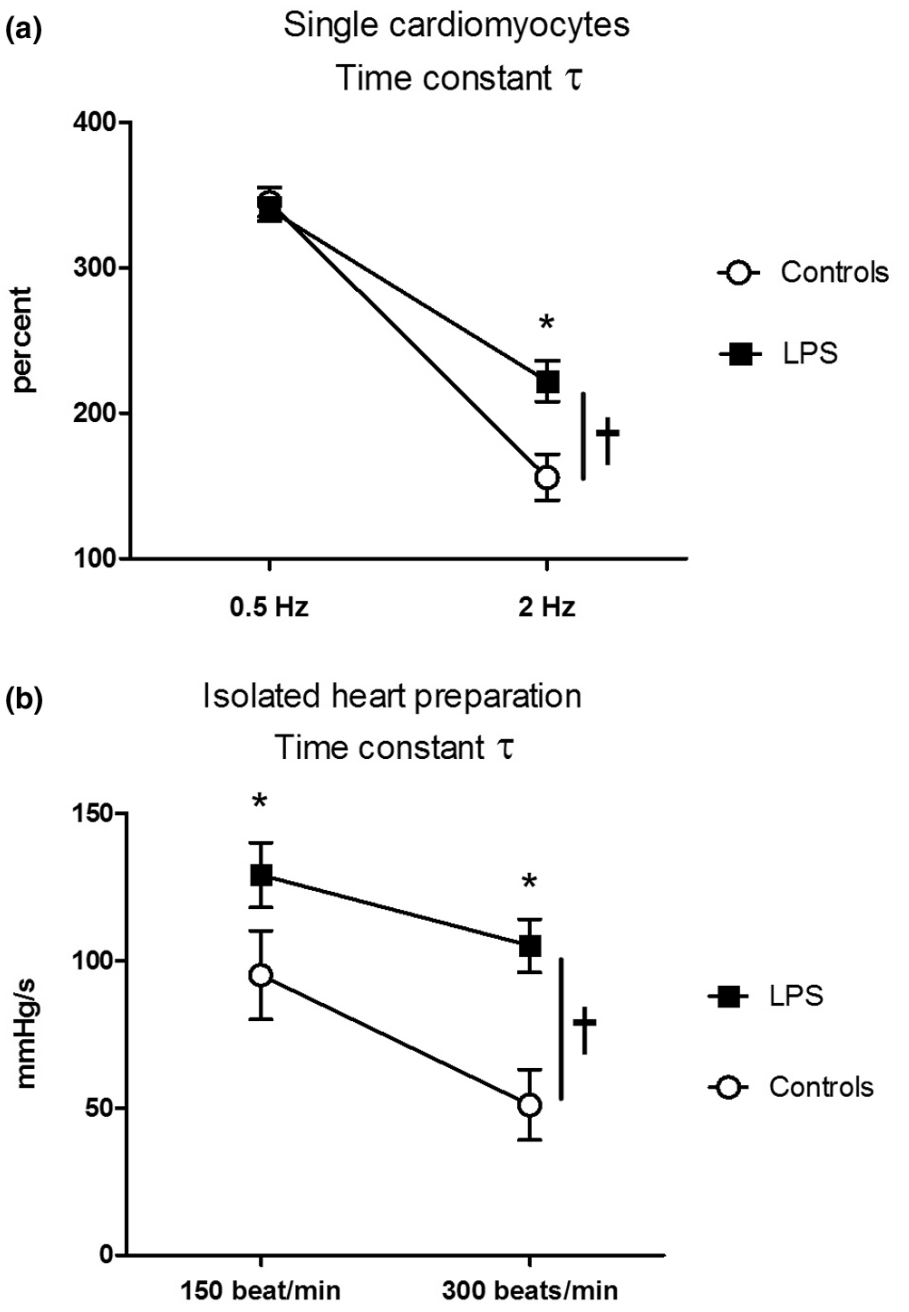
Effects of heart rate changes on frequency-dependent acceleration of relaxation. This was measured in **(a) **single cardiomyocytes (n = 6 per group) and **(b) **isolated heart (n = 8 per group) studies. Results are mean ± standard error of the mean; analysis of variance for repeated measurements on frequency effect, treatment group effect and the interactive effects. * p < 0.05 between control and lipopolysaccharide (LPS) at each frequency; † p < 0.05 between groups across frequency. Overall, LPS resulted in significant different force-frequency dependence in cardiomyocytes and isolated hearts.

In cardiomyocyte and isolated heart preparations, reductions in time constant of relaxation (τ) (Figures 3a,b) and time to 50% relaxation (data not shown) at increasing rate of pacing were lower in LPS-treated rats compared with controls. Echocardiography evaluation at increasing heart rate shown that ratio of Ea change to heart rate change was reduced in LPS-treated rats compared with control rats (0.054 ± 0.026 versus 0.035 ± 0.021 cm/sec/beat, n = 5 rats; p < 0.05). Overall, LPS resulted in significantly different acceleration of relaxation-frequency dependence in cardiomyocytes and isolated hearts.

### Heart calcium regulatory proteins expression, SR phosphatase activity and SR calcium uptake

**Figure 4:**
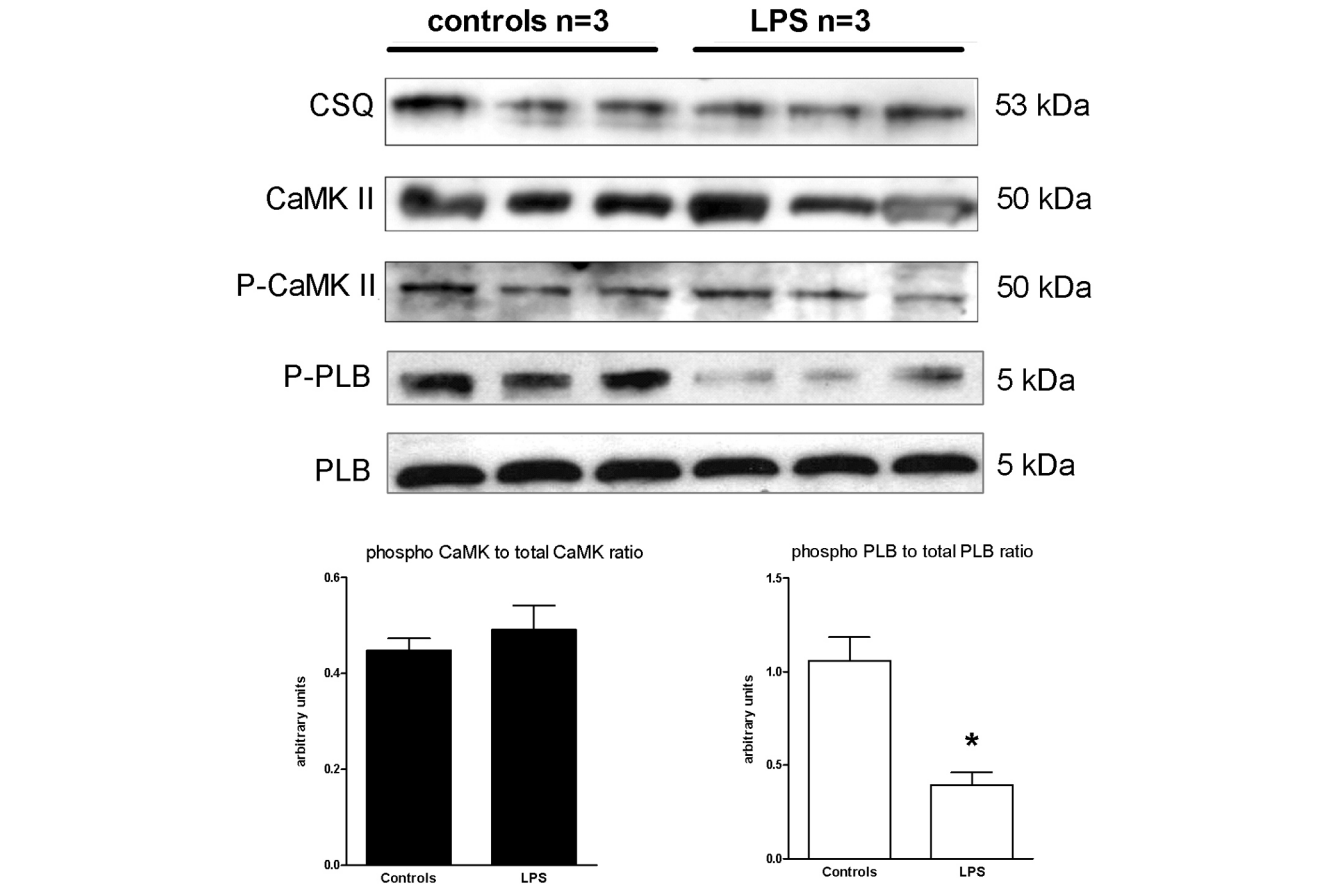
Effects of LPS administration on protein expression in heart tissues. Representative Western blots (upper panel) and statistical analysis (bottom panels) of calsequestrin (CSQ), phosphorylated calcium/calmodulin kinase II (P-CaMKII) and total calcium/calmodulin kinase II (CAMKII), thr17-phospho-phospholamban (P-PLP) and total phospholamban (PLP). Results are presented as mean ± standard error of the mean (n = 6 per group). * p < 0.05 versus controls.

**Figure 5:**
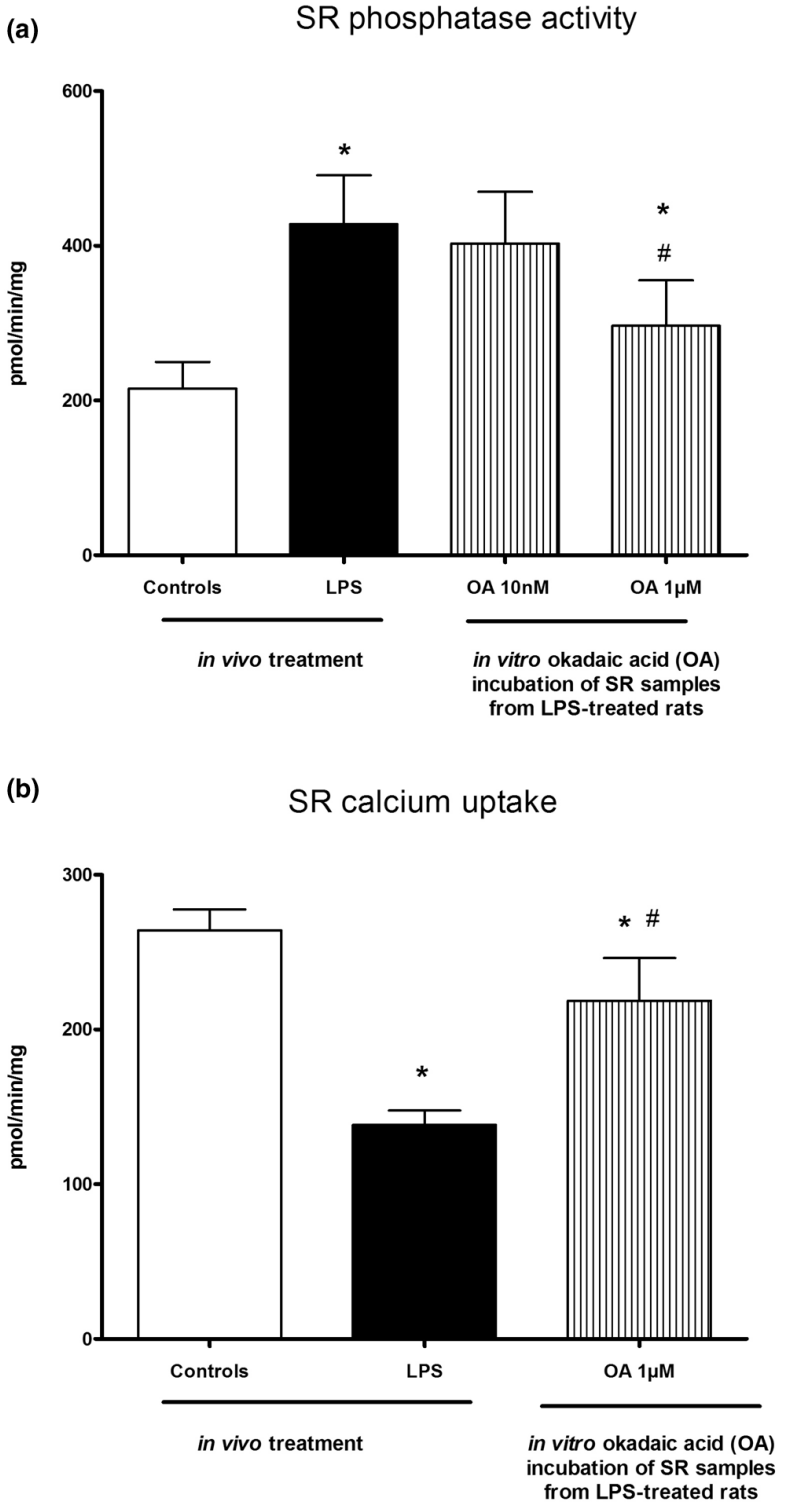
Effects of lipopolysaccharide (LPS) administration on sarcoplasmic reticulum (SR) protein phosphatase activity and SR calcium uptake. Okadaic acid (OA) was used *in vitro *to evaluate differential phosphatase activity. First, heart SR vesicles of sham and LPS-treated rats were prepared. Then, SR vesicles were incubated with OA in order to study phosphatase activity and calcium uptake. Results are presented as mean ± standard error of the mean (n = 6 per group). * p < 0.05 versus controls; # p < 0.05 versus LPS.

LPS treatment was associated with reduction in SR thr17-phosphorylated phospholamban with no changes in total phospholamban protein expression (Figure 4). Compared with controls, LPS challenge did not alter CaMKII activation, that is, phospho-CaMKII to CaMKII ratio (Figure 4). Total protein phosphatase activities were higher in SR vesicles isolated from LPS-treated rats compared with controls rats (Figure 5). Differential phosphatase activities were evaluated by a range of doses of okadaic acid (nM), which inhibits all but PP1 and PP2b phosphatases, and a range of doses of okadaic acid (μM), which inhibits PP1 phosphatases. Incubation of SR samples isolated from LPS-treated rats with okadaic acid at 10 nM had no effects, whereas 1 μM okadaic acid partially reduced phosphatase activity, suggesting that increases in phosphatase activity were only partially related to PP1 and PP2a activities (Figure 5a). Rate of calcium uptake of SR vesicles isolated from LPS-treated rats was reduced compared with controls rats, whereas *in vitro *incubation with 1 μM okadaic acid slightly increased the rate of calcium uptake of SR vesicles isolated from LPS-treated rats (Figure 5b).

### Effects of phosphatase inhibition on FDAR and phospholamban phosphorylation

**Figure 6:**
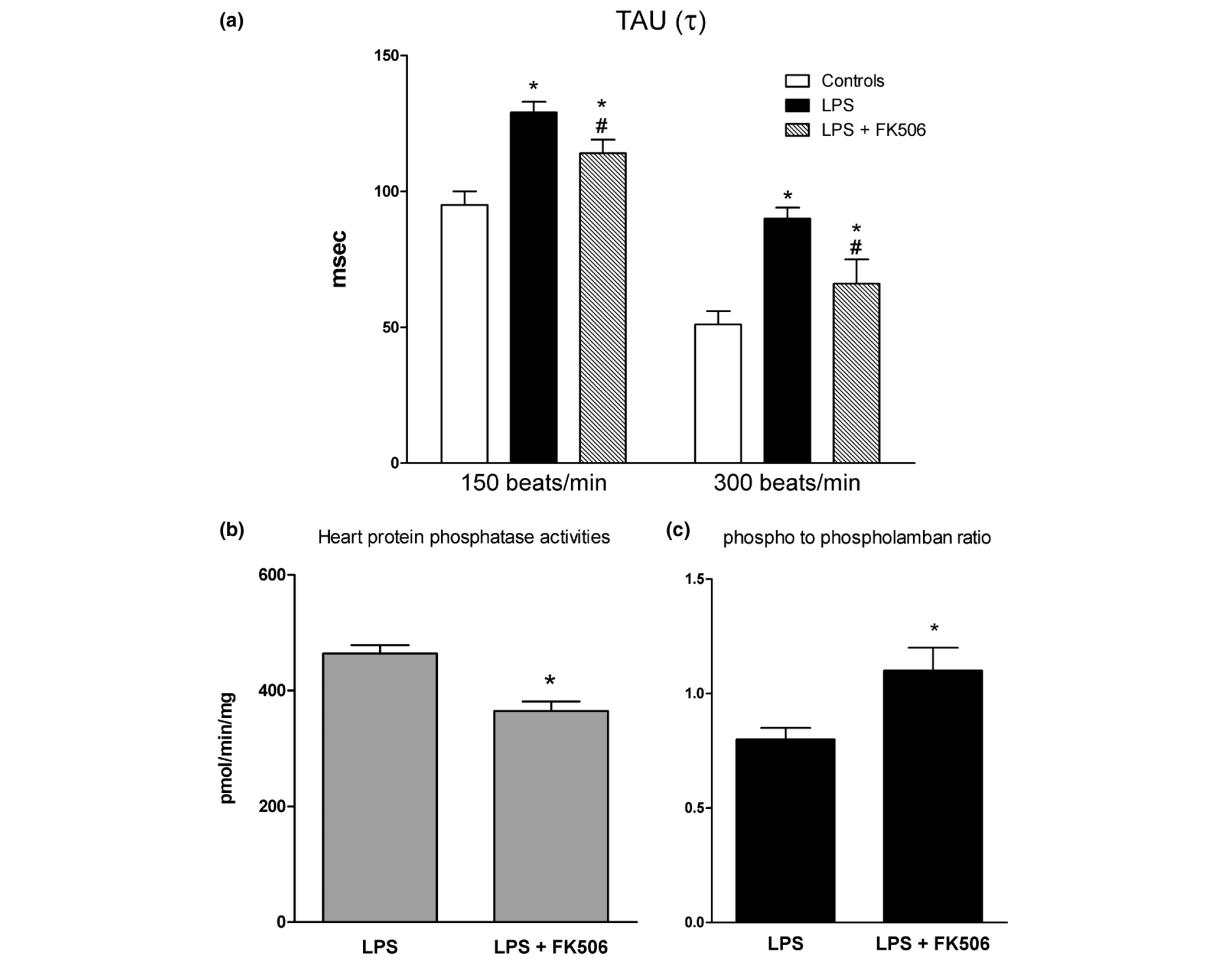
Effects of calcineurin inhibition by tacrolimus (0.01 mg/kg). This was measured in hearts isolated from lipopolysaccharide (LPS)-treated rats on **(a)** time constant of LV relaxation (τ), **(b) **heart protein phosphatase activity and **(c)** phospho-phospholamban to total phospholamban ratio. Results are presented as mean ± standard error of the mean (n = 8 per group). * p < 0.05 versus controls; # p < 0.05 versus LPS.

To evaluate the effects of phosphatase inhibition on FDAR, we evaluated isolated heart characteristics in a new series of experiments in control and LPS rats treated with tacrolimus, a PP2b inhibitor. Tacrolimus in control rats had no effect on LV contractile function, heart phosphatase activities and phospholamban phosphorylation (data not shown). Compared with LPS-treated hearts, tacrolimus did not alter LV contractile performance (dP/dt_max_: 1650 ± 175 mmHg/second in LPS versus 1850 ± 200 mmHg/second in LPS-tacrolimus-treated rats; n = 8 in each group, p > 0.05). Tacrolimus partially restored FDAR (Figure 6a) and normalised heart phosphatase activities and phospholamban phosphorylation (Figures 6b,c) in LPS-treated rats.

## Discussion

Consistent with previous studies [21], our results demonstrated that injection of LPS depresses single cardiomyocyte and LV contractile performance. For the first time, we have demonstrated that LPS-induced intrinsic myocardial dysfunction was frequency dependent with disruption of acceleration of LV relaxation at increasing heart rate. Loss of this fundamental adaptive mechanism that ensures optimal LV filling was accompanied by reduced SR calcium uptake, dephosphorylation of phospholamban and serine/threonine phosphatase activity increases.

In LPS-challenged rats, systolic contractile dysfunction was characterised in single cardiomyocytes, isolated hearts and *in vivo *by echocardiography evaluation. Impairment of heart relaxation associated with LPS was also observed in isolated hearts and *in vivo *preparations. Echocardiography studies further documented relaxation abnormalities as reduction of Ea, a load-independent index of LV relaxation which is impaired in septic patients [22]. Ea reductions in LPS-treated rats were observed in the absence of E/Ea changes; suggesting only minor modification in end-diastolic pressure. The typical positive force-frequency relationship was replaced by an inverted relationship in cardiomyocytes isolated from LPS-treated rat hearts. In contrast, LPS did not alter force-frequency relationships in whole preparations, such as isolated heart and echocardiography, although contractile performance was reduced. FDAR was observed in single cardiomyocytes, isolated hearts and *in vivo *in control rats, whereas LPS blunted this adaptive phenomenon. Because calcium uptake by the SR plays a dominant role in clearance of free cytosolic calcium and thus kinetics of relaxation [6,23], we evaluated calcium handling in SR preparations isolated from controls and LPS-treated rats. We found that SERCA-dependent calcium uptake was reduced in SR preparations of LPS rats, which was associated with reduced phospholamban th-17 phosphorylation and increased serine/threonine protein phosphatase activities. Phospholamban th-17 phosphorylation was specifically studied because increasing heart rates mainly implicate phospholamban phosphorylation at the thr-17 site [6,23]. CaMKII activation, that is the phospho-CaMK to CaMK ratio, was virtually unchanged in the hearts of LPS-treated rat compared with controls. Hence, we speculated that phospholamban dephosphorylation and reduced SR calcium uptake were related to increased phosphatase activity rather than reduction in CaMKII activation. This contention was further supported by the results that *in vitro *SR incubation with okadaic acid, a phosphatase inhibitor, partially restored calcium uptake of SR isolated from LPS-treated rat hearts.

Next, we tested whether phosphatase inhibition *in vivo *would prevent phospholamban dephosphorylation and FDAR perturbations. Okadaic acid, a serine/threonine PP1/PP2a phosphatase inhibitor widely used *in vitro *[24,25], induces hypotension and death *in vivo *[26]. Alternatively, non-specific phosphatase inhibition may be achieved by the use of the calcineurin inhibitor tacrolimus [25]. Because we have previously reported that immunosuppressive doses of tacrolimus (1 mg/kg) have deleterious effects on myocardial function in LPS sepsis [27], low tacrolimus doses were used in this study. We found that 0.01 mg/kg tacrolimus had minimal effects on LV systolic performance and partially restored FDAR responsiveness in LPS-treated rats. Interestingly, tacrolimus normalised heart phosphatase activities and phospholamban phosphorylation in LPS-treated rats. These results are consistent with studies showing that calcineurin inhibition stimulates phospholamban phosphorylation and normalises heart blunted β-adrenoceptor responsiveness, cardiomyocyte time constant of relaxation and rate of calcium decrease in spontaneously hypertensive rats [28].

Our study has important limitations. Our experimental conditions can be considered far removed from the *in vivo *situation, that is, use of an experimental LPS model of sepsis and at frequencies well below the *in vivo *spectrum of the species studied. This can be particularly true in our cardiomyocyte studies, in which pacing rates were 0.5 to 2 Hz. Although rates of pacing were standardised in cardiomyocytes and isolated hearts, heart frequency changes during echocardiography were achieved by increasing doses of volatile anaesthetics, which may alter calcium cycling and myocardial function [29]. For example, halogenated anaesthetics inhibit the post-rest increase of contractile force by impairing the function of SR. Moreover, halothane, which activates the calcium-release channel, can restore the positive shape of the force-frequency relationship in human myocardium, whereas isoflurane and sevoflurane did not change the force-frequency relationship [29]. Hence, volatile (sevoflurane) anaesthesia concentration that was used to lower heart rate, would also impact on systolic and diastolic function *in vivo*. In the present study, immediate effects of heart rate increases on calcium handling were not evaluated. Instead, we tested whether pre-existing calcium handling perturbations induced by LPS would have altered FDAR. Hence, systolic and diastolic changes observed at increased heart rates could be due to pre-existing calcium cycling aberrations and abnormal calcium cycling responses to heart rate increases. We studied calcium handling exclusively in SR preparations, which may not reflect cardiac Ca^2+ ^trafficking. In addition to reduced phospholamban th-17 phosphorylation and increased phosphatase activity, sepsis could also alter FDAR through multiple mechanisms, such as altered beta-adrenergic signalling and cAMP-dependent kinase activity, myofibrillar dysfunction and disturbed nitric oxide signalling. Eventually, tacrolimus, which was used to inhibit protein phosphatase activity, has complex and numerous effects on the regulation of calcium cycling in the heart through its binding to its cellular target, the tacrolimus binding proteins.

## Conclusions

LPS sepsis impairs LV diastolic function and disrupts LV FDAR. Mechanisms involved in these alterations included reduced SR calcium uptake capacities, which may be related to dephosphorylation of phospholamban and protein phosphatase activity increases. We speculated that disruption of LV FDAR, which normally participates in adequate heart cavity filling, may be particularly detrimental in sepsis, a pathological condition typically associated with elevated heart rates and preload dependency.

## Key messages

• LPS sepsis impairs LV diastolic function and disrupts LV FDAR.

• Loss of this fundamental adaptive mechanism that ensures optimal LV filling was accompanied by reduced SR calcium uptake, dephosphorylation of phospholamban and serine/threonine phosphatase activity increases.

## Abbreviations

ANOVA: analysis of variance; CaMKII: calcium/calmodulin protein kinase type II; dP/dt_max_: LV developed pressure first maximal positive derivatives; dP/dt_min_: LV developed pressure first maximal negative derivatives; E: early flow; Ea: early diastolic velocity of the mitral annulus; FDAR: frequency-dependent acceleration of relaxation; LPS: lipopolysaccharide; LV: left ventricle; LVDP: left ventricle developed pressure; LVEDD: left ventricle end diastolic diameter; LVESD: left ventricle end systolic diameter; PW: diastolic posterior wall thickness; SERCA: sarcoplasmic/endoplasmic reticulum calcium ATPase; SR: sarcoplasmic reticulum; SW: septal wall thickness; VTI: velocity time integral.

## Competing interests

The authors declare that they have no competing interests.

## Authors' contributions

OJ and SM performed echocardiographic studies, statistical analyses and drafted the manuscript. SH carried out cardiomyocyte studies, SR preparation and phosphatase activity studies. DM performed isolated heart studies and drafted the manuscript. SL and RN conceived of the study, and participated in its design and coordination and helped to draft the manuscript. All authors read and approved the final manuscript.
